# Evaluation of Real-Time Endogenous Brain-Computer Interface Developed Using Ear-Electroencephalography

**DOI:** 10.3389/fnins.2022.842635

**Published:** 2022-03-24

**Authors:** Soo-In Choi, Ji-Yoon Lee, Ki Moo Lim, Han-Jeong Hwang

**Affiliations:** ^1^Department of Medical IT Convergence Engineering, Kumoh National Institute of Technology, Gumi-si, South Korea; ^2^Department of Electronics and Information Engineering, Korea University, Sejong City, South Korea; ^3^Interdisciplinary Graduate Program for Artificial Intelligence Smart Convergence Technology, Korea University, Sejong City, South Korea; ^4^Department of IT Convergence Engineering, Kumoh National Institute of Technology, Gumi-si, South Korea

**Keywords:** electroencephalography (EEG), ear-EEG, brain-computer interface (BCI), endogenous BCI, test-retest reliability

## Abstract

While previous studies have demonstrated the feasibility of using ear-electroencephalography (ear-EEG) for the development of brain-computer interfaces (BCIs), most of them have been performed using exogenous paradigms in offline environments. To verify the reliable feasibility of constructing ear-EEG-based BCIs, the feasibility of using ear-EEG should be further demonstrated using another BCI paradigm, namely the endogenous paradigm, in real-time online environments. Exogenous and endogenous BCIs are to use the EEG evoked by external stimuli and induced by self-modulation, respectively. In this study, we investigated whether an endogenous ear-EEG-based BCI with reasonable performance can be implemented in online environments that mimic real-world scenarios. To this end, we used three different mental tasks, i.e., mental arithmetic, word association, and mental singing, and performed BCI experiments with fourteen subjects on three different days to investigate not only the reliability of a real-time endogenous ear-EEG-based BCI, but also its test-retest reliability. The mean online classification accuracy was almost 70%, which was equivalent to a marginal accuracy for a practical two-class BCI (70%), demonstrating the feasibility of using ear-EEG for the development of real-time endogenous BCIs, but further studies should follow to improve its performance enough to be used for practical ear-EEG-based BCI applications.

## Introduction

A brain-computer interface (BCI) provides a potential alternative to the normal communication method, which involves languages and body movements, for disabled patients such as those with locked-in syndrome and aphasia (Bauer et al., 1979; Gao et al., 2021; Xu et al., 2021). It translates neuronal brain activity measured invasively or non-invasively into commands for controlling external devices, such as wheelchairs, robot arms, and computers (Hwang et al., 2013a).

Most BCIs have been realized using non-invasive neuroimaging modalities for measuring brain activity on the scalp, such as electroencephalography (EEG), magnetoencephalography (Mellinger et al., 2007), and near-infrared spectroscopy (Power et al., 2012). Among the modalities, EEG has been the most widely used owing to its reasonable cost, portability, and high temporal resolution (Hwang et al., 2013a). However, BCIs based on the traditional scalp-EEG have several disadvantages from the viewpoint of their usability (Looney et al., 2012; Debener et al., 2015; Goverdovsky et al., 2016). They require measurement electrodes to be attached to the scalp with conductive gels, which is time-consuming. Moreover, the electrode attachment time increases with the number of electrodes to be mounted on the scalp. For instance, it generally exceeds 0.5 h for 30 electrodes, which leads to both the subject and operator being exhausted during the preparation for EEG. Furthermore, the electrodes mounted on the scalp are unaesthetic, and subjects need to wash their hair to remove the conductive gels after the BCI experiment. These disadvantages of conventional scalp-EEG-based BCIs render BCI technology difficult to be used outside laboratory environments. As an alternative to the traditional scalp-EEG used in BCIs, some researchers have proposed the use of EEG in which brain activity is measured around or inside the ears; such EEG is termed ear-EEG (Looney et al., 2012; Debener et al., 2015; Bleichner and Debener, 2017; Choi et al., 2018; Kaveh et al., 2020), and measurements can be obtained with a miniaturized and compact hardware system.

The feasibility of using ear-EEG in BCIs has been verified in many previous studies (Bleichner et al., 2015; Bleichner et al., 2016; Fiedler et al., 2016; Fiedler et al., 2017; Choi et al., 2018; Floriano et al., 2018; Wei et al., 2018). Most of the previous studies on ear-EEG-based BCIs have used exogenous paradigms involving external auditory or visual stimuli to evoke stimuli-specific brain activity, such as auditory steady-state response (ASSR) (Kidmose et al., 2012; Looney et al., 2012; Kidmose et al., 2013a,b; Mikkelsen et al., 2015; Goverdovsky et al., 2016; Bech Christensen et al., 2017; Goverdovsky et al., 2017), steady-state visual evoked potential (SSVEP) (Norton et al., 2015; Goverdovsky et al., 2016; Goverdovsky et al., 2017), and event-related potential (ERP) (Kidmose et al., 2012; Bleichner et al., 2015; Debener et al., 2015; Norton et al., 2015; Fiedler et al., 2016; Pacharra et al., 2017). In our previous study (Choi et al., 2018), we showed that an ear-EEG-based BCI can be developed using an endogenous paradigm involving self-modulated brain activity, without any external stimuli, and we classified two different mental states induced during mental arithmetic (MA) and resting state with an average accuracy of about 78%. We also proposed an optimal re-referencing method to improve the signal-to-noise ratio (SNR) of ear-EEG and used it to improve the performance of an endogenous ear-EEG-based BCI (Choi and Hwang, 2019).

Although previous studies have shown the feasibility of using ear-EEG in BCIs, the practical usability of ear-EEG-based BCIs should be verified *via* real-time online experiments that mimic real-life scenarios. To the best of our knowledge, only two studies have introduced online ear-EEG-based BCIs by using a representative exogenous paradigm, SSVEP (Norton et al., 2015; Wang et al., 2015). However, no study has verified the feasibility of an endogenous BCI based on ear-EEG in online environments. To further demonstrate the potential possibility of using ear-EEG on the development of a practically usable BCI, the feasibility of using ear-EEG in developing endogenous BCIs should be also demonstrated, particularly in online experimental environments.

Accordingly, in the present study, we investigated whether an endogenous ear-EEG-based BCI could be reliably implemented with reasonable performance in real-world applications. We developed an online endogenous ear-EEG-based BCI and tested it on two days to investigate not only its reliability as a real-time endogenous ear-EEG-based BCI, but also its test-retest reliability. In the experiment, three mental tasks were employed: MA, mental singing (MS), and word association (WA). An offline experiment was first conducted to determine the best pair of mental tasks for each subject, and online experiments were then conducted on 2 days using individually selected best pairs of mental tasks.

## Materials and Methods

### Subjects

Fourteen healthy subjects (average age: 25.57 ± 1.70 years; eight males and six females) participated in this study. All of them had normal or corrected-to-normal vision and hearing. None of them reported previous neurological, psychiatric, or other related diseases that could have affected the outcomes of this study. To minimize the impact of the subjects’ physical condition on the experiment, the subjects were asked to sleep for at least 6 h on the day preceding the experiment and to avoid alcohol intake for at least 24 h before the experiment. Information regarding the detailed procedure of this study was provided before the experiment. All subjects gave informed consent prior to the beginning of the experiments and received monetary compensation after the experiment. This study was approved by the Institutional Review Board of the Kumoh National Institute of Technology (No. 6250), and was performed in accordance with relevant guidelines and regulations.

### Ear-Electroencephalography (EEG) Measurement

**FIGURE 1:**
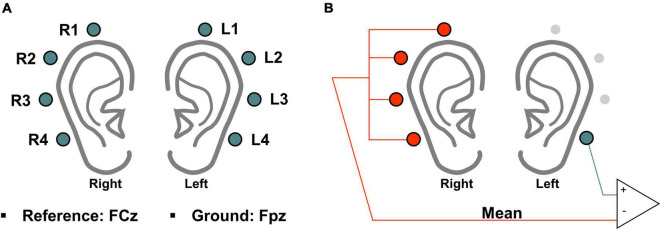
**(A)** Schematic of electrode positions used to record ear-EEG data. **(B)** Schematic sketch of the re-referencing method used to remove the impact of the original reference electrode (FCz). Re-referencing was performed by subtracting the mean value of the opposite ear’s electrodes from the value of an electrode of interest for every time point.

The experiment was conducted in a soundproof room, and the subjects were seated in comfortable armchairs in front of a 21-in. monitor (LG, 24MP58VQ, Seoul, South Korea) and binaural speakers (Britz, BR-1000A, Cuve Black 2, Paju, South Korea). Ear-EEG data were recorded using eight electrodes attached behind the ears (four electrodes for each ear), as shown in Figure 1A, at a sampling rate of 1,000 Hz (actiCHamp, Brain Products GmbH Ltd., Gilching, Germany). In accordance with the international 10–20 system, the reference and ground electrodes were attached at the FCz and Fpz positions, respectively, but the reference effect was removed by re-referencing with only ear-EEG (Figure 1B), which is explained later in this paper. The impedance was maintained below 10 kΩ throughout the experiment.

### Experimental Paradigm

#### Offline Experiment Conducted on Day 1

The objective of the offline experiment was to select the best pair of mental tasks for each subject, which was used in the subsequent online experiments conducted on two different days. The offline experiment was hence performed by considering the following three mental tasks:

(i) MA: Continuously subtracting a single-digit number (between 5 and 9) from a three-digit number (e.g., 477 − 8 = 469, 469 − 8 = 461, …), with both numbers being randomly presented (Shin et al., 2016; Choi et al., 2018).
(ii) WA: Generating words beginning with a letter provided to the subjects in their native language (Korean; e.g., Apple, Arrow, Aerospace, … for “A”) (Friedrich et al., 2012, 2013). The subjects had to generate as many words as they could.
(iii) MS: Mentally singing the English alphabet song from A to Z at a constant speed of 1 Hz, which induced relatively lower cognitive load compared with MA and WA (Shin et al., 2016).

**FIGURE 2:**
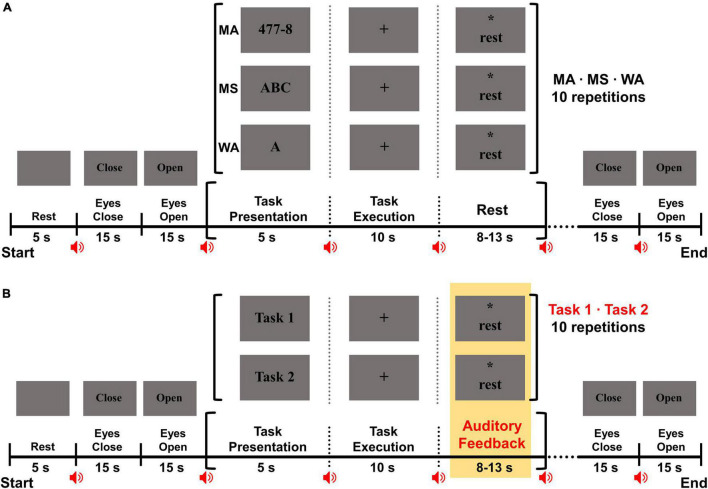
**(A)** Schematic of the offline experimental paradigm. At the beginning of each session, a 5 s rest period was provided for preparing for the upcoming task. Before and after performing three mental tasks, the eyes were closed and opened for 15 s each to check the reliability of ear-EEG data related to alpha activity changes. A single trial comprised a 5 s task presentation period, a 10 s task execution period, and an 8 to 13 s rest period. During a task presentation period, a mental task that was to be performed was displayed for 5 s (e.g., 477 – 8 for MA, the string “ABC” for MS, and a Korean character for WA). During a task execution period, subjects were instructed to perform an indicated mental task for 10 s while focusing on a black fixation cross displayed at the center of a screen, to minimize eye movements. During a rest period, the word “rest” was presented along with an asterisk on the screen, and the subject was instructed to take a break without any thought and movement while staring at the asterisk. A short beep was presented at every screen transition (red speaker icons) to explicitly indicate the transition. **(B)** Schematic of the online experimental paradigm. The online experiment paradigm was identical to that of the offline experimental paradigm, except that an individually selected best pair of mental tasks was used for each subject and auditory feedback was provided on the basis of real-time classification results after task execution.

Figure 2A shows the experimental paradigm of the offline experiment. Each subject completed five experimental sessions. At the beginning of each session, a blank image was presented for 5 s, during which the subjects were instructed to relax and prepare to act according to the upcoming instruction. After the rest period, eyes-closed (EC) and eyes-open (EO) tasks were sequentially performed, each for 15 s, with the aim of verifying the reliability of ear-EEG measurements based on alpha activity changes (Looney et al., 2012; Goverdovsky et al., 2016; Choi et al., 2018; Choi and Hwang, 2019). Subsequently, the three mental tasks were randomly performed, with each task being performed 10 times, in a single session. A single trial comprised task presentation for 5 s, subsequent task execution for 10 s, and a variable rest period that ranged from 8 to 13 s. During the task presentation period, a combination of three-digit and one-digit numbers for MA (e.g., 477 – 8), a single letter for WA (e.g., “A”), or the string “ABC” for MS was randomly presented on the monitor. The corresponding mental task was performed for the following 10 s, during which time the subjects gazed at a fixation mark presented at the center of the monitor to minimize eye movements. During the variable rest period, the word “rest” and an asterisk were presented on the monitor, and the subjects were instructed to gaze at the asterisk with a blank mind to minimize eye movements. The three mental tasks were randomly presented in a session, and each session was ended with EC and EO tasks. Each subject completed five sessions, performing 50 trials for each of the three mental tasks. There was a break of 3 to 5 min between the sessions to allow the subjects to rest.

#### Online Experiment Conducted on Day 2 and 3

On the basis of the classification accuracies of all combinations of the three mental tasks performed in the offline experiment (i.e., MA vs. MS, MA vs. WA, and MS vs. WA), we selected the best pair of mental tasks for each subject and used them for the following online experiments performed on two different days. The online experiment comprised three training and two test sessions, where we collected training data (30 trials for each task) using an individually selected best pair of mental tasks to construct an online classifier and we tested the classifier for new inputs (test data: 20 trials for each task) in real-time, respectively. The experimental paradigm of the online experiments was identical to that of the offline experiment, except that the best pair of mental tasks was employed for each subject and real-time feedback was provided in the two test sessions immediately following the task execution period, on the basis of the classification results (Figure 2B). In the first online experiment conducted on day 2, three training sessions were first performed. No feedback was provided during mental tasks in these sessions, whereas auditory feedback was provided on the basis of real-time classification results in the two test sessions following the task execution period. The experimental paradigm of the online experiment conducted on day 3 was identical to that used for the first online experiment conducted on day 2, except that the two test sessions were repeated thrice independently using three different online classifiers. We tested these three classifiers that were constructed using different training data sets, namely, the data set obtained on day 2, that acquired on day 3, and the combination of these two data sets, to investigate the impact of different training data on their classification performance.

### Ear-Electroencephalography (EEG) Data Analysis

#### Preprocessing

Data analysis was performed using MATLAB (MathWorks, Natick, MA, United States) along with the EEGLAB (Delorme and Makeig, 2004) and BBCI toolboxes (Blankertz et al., 2016), and the same analysis method was applied to the ear-EEG data measured in both offline and online experiments. Ear-EEG data were first band-pass filtered from 1 to 50 Hz using a zero-phase third-order Butterworth filter, and then down-sampled to 200 Hz to reduce the computation time. As the ear-EEG data were recorded using a reference at FCz, for the removal of the impact of the original reference electrode, all ear-EEG data were re-referenced using the mean value of the ear-EEG channels on the opposite ear because of the superior SNR of the re-referencing method (Choi and Hwang, 2019). Then, 10 s-epochs based on the task onset were extracted for each of the three mental tasks for classification. Figure 1B shows an example of the re-referencing method for one channel denoted by turquoise blue color on the left ear, and each of all channels were re-referenced by the same method.

#### Classification

A multiband common spatial pattern (CSP) was used to determine discriminative features for classification in five frequency bands (δ-band: 1–3 Hz, θ-band: 4–7 Hz, α-band: 8–13 Hz, β-band: 14–29 HZ, and γ-band: 30–50 Hz) (Ramoser et al., 2000; Lemm et al., 2005). A multiband CSP was independently applied to the 10-s epochs of the three mental tasks, respectively, for each of the five frequency bands, where the log-variances of the first and last CSP components were extracted for each frequency band as classification features. Shrinkage linear discriminant analysis (sLDA; Peck and Ness, 1982; Schäfer and Strimmer, 2005; Blankertz et al., 2011) was used as a classifier. Offline classification accuracies were obtained using 10-fold cross-validation for all possible pairs of the three mental tasks (MA vs. WA, MA vs. MS, and WA vs. MS) and they were used to select the best pair of mental tasks for each subject on day 1. Online classification accuracies were estimated using real-time outputs obtained immediately after performing a single trial in the two test sessions on days 2 and 3. As mentioned, two test sessions were performed thrice independently in the online experiment conducted on day 3. In the sessions, three different sLDA classifiers were constructed using different training data (data obtained on day 2, data acquired on day 3, and the combination of data obtained on these 2 days), and the online classification accuracies were independently estimated for each of the three classifiers.

#### Statistical Test

To investigate the feasibility of constructing an ear-EEG-based online BCI, we compared the online classification performance of our ear-EEG-based BCI with a theoretical 95% confidence limit of a chance accuracy on the basis of the number of trials for a two-class BCI (e.g., 59.61% for 50 trials of each task) (Müller-Putz et al., 2008). Furthermore, we employed a non-parametric statistical method, the Friedman test, for performing multiple comparisons with the Wilcoxon signed-rank test for *post hoc* in terms of the classification performance since the number of samples was insufficient (<30) for using parametric methods (i.e., RM-ANOVA) (Hesterberg, 2015).

#### Event-Related (de)Synchronization

To visually inspect task-specific brain activity in terms of event-related (de)synchronization (ERD/ERS), event-related spectral perturbation (ERSP) of each mental task was estimated for each epoch extracted from −2 to 10 s on the basis of the task onset for each channel and subject (baseline period: −2 to 0 s), and ERSPs were averaged over all subjects (Friedrich et al., 2012). To quantitatively investigate changes in ERSP values over three experimental days, we estimated ERSP values of three experimental days for five frequency bands (δ-band: 1–3 Hz, θ-band: 4–7 Hz, α-band: 8–13 Hz, β-band: 14–29 HZ, and γ-band: 30–50 Hz) for each of three mental tasks.

## Results

### Offline Experimental Results

Table 1 shows the classification accuracies for each pair of the three mental tasks and the best pairs of mental tasks for each subject. The average classification accuracies of MA vs. MS, MA vs. WA, and MS vs. WA were 73.1 ± 13.9%, 69.5 ± 14.2%, and 69.5 ± 12.5%, respectively. The mean classification accuracy of the best pairs of mental tasks for each subject was 77.7 ± 10.9% (last column of Table 1), which was similar to that of our previous study (Choi and Hwang, 2019). Statistical test results showed that the mean classification accuracy of individually selected best pairs of mental tasks was significantly higher than those of MA vs. WA and MS vs. WA (Friedman: χ2(3) = 15.88, *p* = 0.0012, best pair > MA vs. WA = MS vs. WA), whereas the mean classification accuracy of MA vs. MS was not statistically different from the others. The combination of MA and MS was mostly selected as the best pair of mental tasks: MA vs. MS = 7, MA vs. WA = 4, and WA vs. MS = 3.

**TABLE 1:** Classification accuracies (%) of each pair of mental tasks for each subject in the offline experiment. The bold-font indicates the classification accuracies for the best pair of mental tasks for each subject.

	MA vs. MS	MA vs. WA	MS vs. WA	Best pair
Sub 1	**55.2**	55.1	42	55.2
Sub 2	76.7	**80.6**	64.7	80.6
Sub 3	**80.8**	65.3	70.6	80.8
Sub 4	63.7	70.2	**75.6**	75.6
Sub 5	51.4	67.3	**77.6**	77.6
Sub 6	**97.7**	96.8	93.6	97.7
Sub 7	55.8	54.2	**65.4**	65.4
Sub 8	**87.1**	48.4	83.5	87.1
Sub 9	**71.1**	65.8	58.7	71.1
Sub 10	81.4	**81.5**	72.2	81.5
Sub 11	**91.5**	83	79.1	91.5
Sub 12	74.9	**82.9**	67.5	82.9
Sub 13	**69.6**	51.2	61.7	69.6
Sub 14	66.8	**71.2**	60.7	71.2
**Average**	**73.1 ± 13.9**	**69.5 ± 14.2**	**69.5 ± 12.5**	**77.7 ± 10.9**

### Online Experimental Results

**FIGURE 3:**
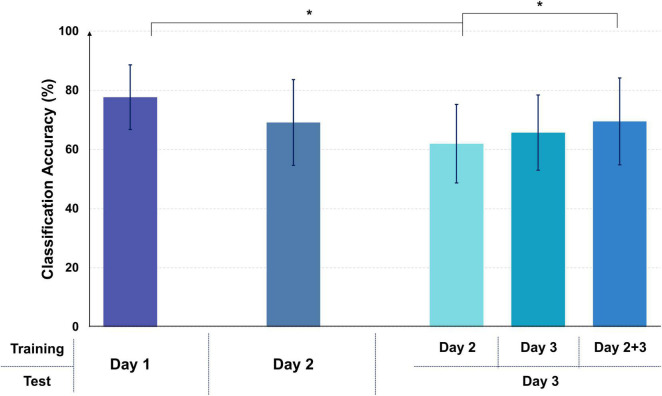
Mean classification accuracies of the offline experiment (day 1) and the two online experiments (days 2 and 3). The mean offline classification accuracy was obtained by averaging the offline classification accuracies for the best pairs of mental tasks for each subject (day 1). The mean classification accuracies of day 3 were obtained using three different training data sets (day 2, day 3, and day 2 + day 3). An asterisk indicates a statistically significant difference.

Figure 3 shows the mean classification accuracies for the two online experimental days (days 2 and 3), along with the best mean offline classification accuracy shown in Table 1 (day 1: 77.7 ± 10.9%). The mean online classification accuracies for days 2 and 3 were 69.1 ± 14.5% and 65.7 ± 12.7% (when only training data measured on day 3 was used), respectively, which were not statistically different from the best mean offline classification accuracy, despite the reduced performances. The mean online classification accuracy for day 3 decreased when the training data obtained on a different day (day 2) was used to build a classifier (61.9 ± 13.2%); by contrast, it increased when a combination of the training data acquired on two days (day 2 + day 3) was used (69.5 ± 14.7%). Despite the inter-experimental variability of the classification accuracies, all the mean online classification accuracies were higher than the theoretical 95% confidence limit of a chance accuracy (59.61%), and a classification accuracy of nearly 70% (marginal classification accuracy for a practical two-class BCI) was obtained for two online experimental days (for day 3, when a combination of training data obtained on days 2 and 3 was used).

### Event-Related (de)Synchronization Maps

**FIGURE 4:**
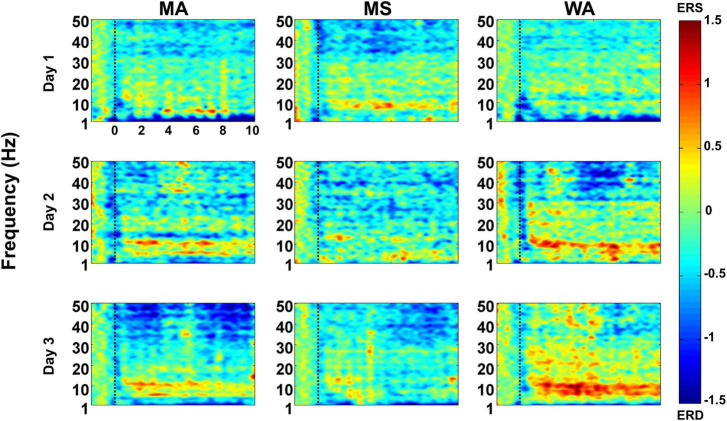
ERD/ERS pattern maps averaged over all channels and subjects over the three experimental days. The x-axis indicates the task period between −2 to 10 s on the basis of the task onset, and the y-axis represents the frequency ranging from 1 to 50 Hz. The task onset at *t* = 0 s is marked by a dotted line.

Figure 4 shows the grand-average ERD/ERS maps of all subjects for each mental task over the 3 days; the maps were obtained by averaging all channels. Overall, for all three mental tasks, on day 1 (offline experiment), widespread ERS was observed in a middle frequency band (7–30 Hz), while relatively strong ERD was observed in the other frequency band (first row of Figure 4). However, despite the similar ERD/ERS patterns, each mental task showed relatively distinct ERD/ERS patterns, making it possible to distinguish each of the tasks, such as relatively stronger δ- and θ-ERD for MA than MS while stronger γ-ERD for MS than MA. Moreover, relatively small changes were observed in ERD/ERS for WA compared with MA and MS. The ERD/ERS pattern for MA— α- and β-ERS and γ-ERD—tended to become stronger from the first experimental day to the last experimental day (day 1 → day 2 → day 3 in the first column of Figure 4). Furthermore, WA particularly started to show its unique ERD/ERS pattern from the first online experiment (day 2), namely, widespread strong ERS over the entire frequency band along with strong γ-ERD. However, small changes in ERD/ERS were observed for MS over the three experimental days. The quantitative analysis results for ERD/ERS pattern changes over three experimental days are provided with respect to five frequency bands (δ-band: 1–3 Hz, θ-band: 4–7 Hz, α-band: 8–13 Hz, β-band: 14–29 Hz, and γ-band: 30–50 Hz) for each of three mental tasks in Supplementary Figure 1.

## Discussion

Recently, ear-EEG has gained considerable attention for its potential use in the development of BCIs owing to its high usability and portability for brain activity measurement, despite its relatively low performance compared with scalp-EEG-based BCIs (Pacharra et al., 2017; Denk et al., 2018; Kaongoen et al., 2021). Most of the previously proposed ear-EEG-based BCIs have involved exogenous paradigms, such as SSVEP (Norton et al., 2015; Goverdovsky et al., 2016; Goverdovsky et al., 2017), ASSR (Mikkelsen et al., 2015; Bech Christensen et al., 2017), and ERP (Looney et al., 2012; Debener et al., 2015; Fiedler et al., 2016; Pacharra et al., 2017), and their feasibility has been demonstrated *via* offline experiments in general. However, the feasibility of using ear-EEG in the development of endogenous BCIs should be also demonstrated, especially in online experimental settings that mimic real-world scenarios, in order to extend the feasibility of using ear-EEG in the development of BCIs. In this study, we investigated the feasibility of an ear-EEG-based BCI involving self-modulated EEG by using an endogenous paradigm in an online environment, and we demonstrated that a real-time endogenous BCI can be implemented using ear-EEG, despite its decreased classification performance compared with that in offline experiments.

The offline classification accuracy of each mental task pair differed among the subjects. The mean offline classification accuracy for the best pair of mental tasks (77.7 ± 10.9%) was significantly higher than those of the other two pairs of mental tasks (Table 1), namely MA vs. WA (69.5 ± 14.2%) and MS vs. WA (69.5 ± 12.5%), indicating the importance of using individualized mental tasks for the development of reliable BCIs (Hwang et al., 2014). The mean online classification accuracies for days 2 and 3 were lower by about 8 and 12% (day 2: 69.1 ± 14.5%; day 3: 65.7 ± 12.7%), respectively, compared with that of the offline experiment (77.7 ± 10.9%). The lower classification performance in the online experiment was probably because of the difference in the experimental environment between the training and test sessions; the difference depended on whether real-time feedback regarding classification results was provided (Shenoy et al., 2006). In general, the environmental difference between offline and online experiments gave rise to the inherent non-stationarity of EEG data, thereby resulting in a shift in the data distributions in feature space and ultimately degrading the BCI classification performance (Shenoy et al., 2006). On the other hand, real-time feedback provided in the online experiment reduced the impact of inter-day (session) variability on classification performance because the subject could adapt a given classifier by chaining a control strategy based on real-time feedback (Hwang et al., 2017); two classifiers trained using the EEG data measured on days 2 and 3, respectively, showed a small difference of classification accuracy when testing the EEG data measured on day 3 (61.9 ± 13.2% vs. 65.7 ± 12.7%). Despite the reduced classification performance in the online experiment, we obtained a meaningful mean classification accuracy of nearly 70%, which is a marginal classification accuracy for practical communication in two-class BCIs. Thus, we demonstrated that ear-EEG could be used to realize real-time endogenous BCIs. Nevertheless, the overall classification performance of our proposed ear-EEG-based BCI should be improved in order to increase the reliability of an ear-EEG-based endogenous BCI. Because the classification performance drop was partly compensated when the amount of training data was increased on day 3 (65.7 ± 12.7% → 69.5 ± 14.7% when a combination of training data of days 2 and 3 was used), it is expected that the classification accuracy will naturally increase and stabilize as a user uses a BCI over several days, owing to the cumulative data. Another approach to prevent the classification performance drop in the online experiment would be to introduce adaptive algorithms that use new data measured in real-time feedback sessions for classifier adaptation (Blankertz et al., 2007). We intend to work toward enhancing the performance of the proposed real-time ear-EEG-based BCI with the objective of developing reliable practical ear-EEG-based BCIs.

In this study, we employed three mental tasks (MA, MS, and WA) that have been widely used in previous BCI studies, and they showed somewhat overlapping but unique ERD/ERS patterns. MA showed relatively strong ERS in α- and β-bands and widespread ERD in a high frequency band (γ-band), which were similar to observations made in our previous studies (Choi et al., 2018; Choi and Hwang, 2019). Interestingly, the ERD/ERS pattern of MA became more dominant with the passage of time; from a neurophysiological viewpoint, this can be attributed to learning through real-time feedback in the online experiment, which led to better facilitation of brain activity (Duan et al., 2021). The ERD/ERS pattern of WA somewhat overlapped with that of MA in terms of α- and β-ERS with γ-ERD, but was also different from MA (e.g., relatively stronger θ- and α-ERS and shorter period of γ-ERD compared with MA). In particular, θ- and α-ERS became considerably stronger over the days for WA, which could be attributed to the learning, similar to MA. Unlike MA and WA, however, MS did not show significant changes in ERD/ERS patterns over the days, which could be explained by MS being the mental task originally designed to induce low cognitive load without intensively involving the brain resources during the mental task (Choi et al., 2018; Choi and Hwang, 2019). The qualitative results shown in Figure 4 were also confirmed by the quantitative results shown in Supplementary Figure 1, such as an increasing trend of γ-ERD for MA and that of α-ERS for WA over three experimental days. Although the unique ERD/ERS patterns of each mental task tended to become stronger over the days because of learning through real-time feedback, as mentioned above, the overall classification performance rather decreased from day 1 to day 3, which might be because of the overlapping ERD/ERS patterns between the mental tasks despite their unique ERD/ERS patterns, such as the relatively strong ERS and ERD in the low- and high-frequency bands, respectively, as well as increased non-stationarity of EEG data between the training and test sessions in the online experiment. Therefore, it is necessary to develop more advanced algorithms that can fully utilize task-specific ERD/ERS patterns that change over the days, to improve the overall performance of endogenous ear-EEG-based BCIs.

Because BCI technology was introduced to help paralyzed patients to communicate with the outside world, BCI spellers have been widely developed, mainly using exogenous paradigms, such as SSVEP (Hwang et al., 2013b; Lim et al., 2015) and ERP (Gong et al., 2020; Miao et al., 2020). As an alternative to exogenous BCI spellers that require auditory or visual stimuli, imagined speech has been also actively studied for communication purposes. The auditory cortex close to the ears is responsible for speech and has shown task-specific brain activity during imagined speech (Kaongoen et al., 2021). In this study, we used three mental tasks that have been most widely used in BCI studies, but imagined speech might be the mental task most suitable for implementing ear-EEG-based BCIs since ear-EEG can capture task-specific brain activity generated from the auditory cortex more reliably compared with other brain areas owing to the adjacency effect. Therefore, it would be interesting to investigate the feasibility of using imagined speech in ear-EEG-based endogenous BCIs.

## Data Availability Statement

The raw data supporting the conclusions of this article will be made available by the authors, without undue reservation.

## Ethics Statement

The studies involving human participants were reviewed and approved by the Institutional Review Board of the Kumoh National Institute of Technology. The patients/participants provided their written informed consent to participate in this study.

## Author Contributions

S-IC, KL, and H-JH designed the experiment and wrote the manuscript. S-IC and J-YL acquired the data. S-IC and H-JH performed the data analysis. All authors contributed to the article and approved the submitted version.

## Conflict of Interest

The authors declare that the research was conducted in the absence of any commercial or financial relationships that could be construed as a potential conflict of interest.

## Publisher’s Note

All claims expressed in this article are solely those of the authors and do not necessarily represent those of their affiliated organizations, or those of the publisher, the editors and the reviewers. Any product that may be evaluated in this article, or claim that may be made by its manufacturer, is not guaranteed or endorsed by the publisher.
