# Supplementary material for: Evaluation of Real-Time Endogenous Brain-Computer Interface Developed Using Ear-Electroencephalography
Source: Front Neurosci. 2022 Mar 24;16:842635. doi: 10.3389/fnins.2022.842635 (PMC8987155; doi:10.3389/fnins.2022.842635)
Supplement: Supplementary file 1 [file Data_Sheet_1.docx]

Supplementary Material


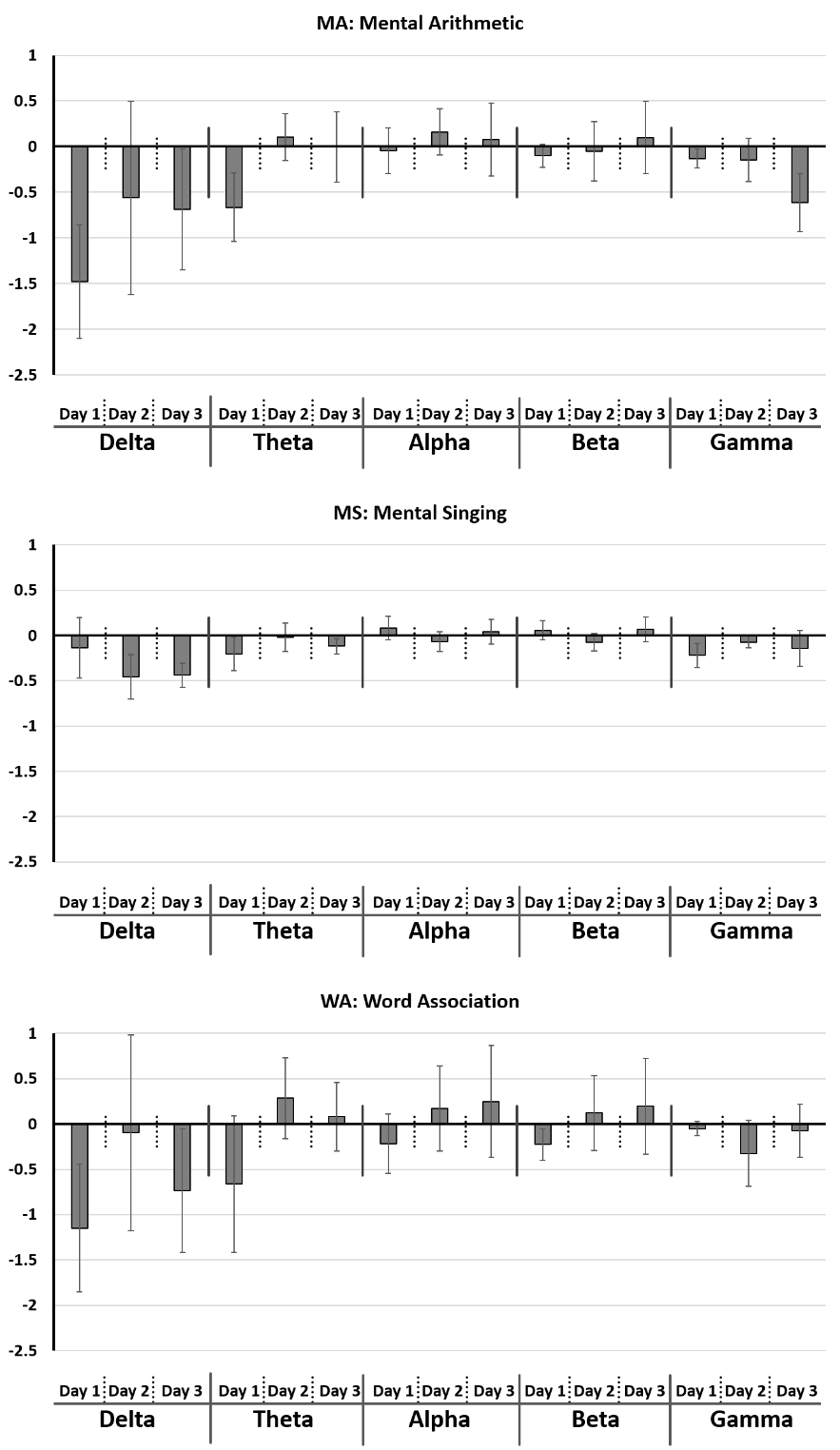


**Supplementary Figure 1.** Grand-average ERSP values of three experimental days for five frequency bands (δ-band: 1‒3 Hz, θ-band: 4‒7 Hz, α-band: 8‒13 Hz, β-band: 14‒29 HZ, and γ-band: 30‒50 Hz) for each of three mental tasks. No significant difference was observed between the ERSP values of three experimental days for all frequency bands and mental tasks (Friedman test *p* > 0.05). Error bars indicate standard errors.
